# The 2^nd^ Conference and Workshop of The Cancer Genome Atlas (TCGA) in India: Towards Team Science for Multi-omics Cancer Research in South Asia

**DOI:** 10.3332/ecancer.2021.ed111

**Published:** 2021-05-25

**Authors:** Santosh Dixit, Anguraj Sadanandam

**Affiliations:** 1 Centre for Translational Cancer Research, Pune, India; 2 Centre for Global Oncology, The Institute of Cancer Research, London, United Kingdom; 3 Division of Molecular Pathology, The Institute of Cancer Research, London, United Kingdom

**Keywords:** cancer genomics, multi-omics cancer research, open-source databases, india, training, conference report, TCGA, ICGA, global oncology

## Abstract

The Cancer Genome Atlas (TCGA) is a landmark cancer genomics program that molecularly characterized over 20,000 primary cancer and matched normal samples spanning 33 cancer types. On similar lines, the establishment of an **‘Indian Cancer Genomics Atlas (ICGA)’** has been initiated in 2019–2020 by a consortium of key stakeholders in India led by Council for Scientific and Industrial Research (CSIR), Government of India and several reputed governmental agencies, cancer hospitals, academic institutions, and private sector partners. In parallel, Bangladesh Medical Research Council (BMRC) has announced the launch of ‘Bangladesh Cancer Genome Atlas (BCGA) project with support from the ICGA teams. Teams from United States - National Cancer Institute (NCI) office of TCGA and Centre for Global Oncology, Institute of Cancer Research, London, United Kingdom are interested in extending their collaborations to these large-scale initiatives by acting as knowledge partners.

With this background, an online version of the 2^nd^ TCGA conference and workshop in India was organised with the theme of ‘Towards Team Science for Multi-omics Studies in South Asia’ on December 3–5, 2020. Over 1,500 delegates comprising of onco-clinicians, basic researchers, bioinformaticians, geneticists, translational researchers, big-data and machine-learning scientists, bioethicists and regulatory experts from across the globe attended the event. The conference agenda focused on the vision, design and plans of the ICGA project with regards to common standard operating protocols (SOPs), operations, logistics, bioethics, policy and governance models. More importantly, conference sessions were planned around the central theme of building a culture of team science for undertaking mega-cancer research projects in India and neighbouring countries. Experts from the globe deliberated on the latest technical aspects of data/biospecimen/multi-omics studies and applications of Precision Oncology in clinical cancer management.

## Introduction

Over the last 15 years, The Cancer Genome Atlas (TCGA) program has characterized over 20,000 primary cancers and matched normal samples across 33 cancer types. By networking interdisciplinary researchers from across the world, TCGA has generated a massive 2.5 petabytes of genomic, epigenomic, and transcriptomic data. These data, not least through their publicly availability, have already led to significant and potential translatable benefits in the diagnosis, treatment, and prevention of cancer. The importance of the project and its participants was recently recognized with the prestigious 2020 American Association of Cancer Research ‘Team Science Award.’

There are an estimated 800,000 new cancers cases in India each year [1] and, recognizing this individual and societal burden, a number of initiatives have been established to expand India’s genomics capacity. The Department of Biotechnology (DBT), Government of India (GoI) initiated the ‘GenomeIndia Project’ in 2020 to sequence 10,000 healthy individuals. India is also a partner in the International Cancer Genome Consortium (ICGC), where it has a special focus on gingivo-buccal cancer [2]. In 2020, the Council of Scientific and Industrial Research (CSIR), GoI committed to establishing Cancer Consortia to sequence Indian cancer patients. These initiatives will be rolled out over the coming years in partnership with various Governmental and non-Governmental agencies. Learning from the successes and challenges of TCGA, it is imperative to establish a collaborative infrastructure in India to facilitate the sharing and analyses of these big data.

## The Indian Cancer Genome Atlas (ICGA): mission, structure, and strategy

To this end, the ‘Indian Cancer Genome Atlas (ICGA)’ was initiated in 2020 by forming a national consortium of government agencies, cancer hospitals, academic institutions, and private sector partners in India, led by the Council of Scientific and Industrial Research (CSIR). The ICGA will be a long-term collaborative effort between cancer scientists, basic researchers, onco-clinicians, data scientists, and technology providers to facilitate precision medicine and improve translational cancer research in India.

The ICGA’s mission is to create an indigenously developed, open-source, comprehensive database of multi-omics profiles of all possible cancers in Indian populations. The goal is the genomic, transcriptomic, epigenetic, and proteomic characterization of Indian cancers using advanced, next-generation multi-omics technologies. High quality meta-data of cancer patients and their biospecimens (blood, cancer tissues) will be carefully and ethically collected from across the country. After standardized multi-omics profiling, data will be curated and analyzed in clinicopathological contexts, with the curated databases open-sourced to the Indian and global cancer research communities. To facilitate the uniformity, sanctity, and integrity of the meta-data and biospecimens collected from across India, a pan-India consortium of partners will operate as a ‘hub-and-spoke’ model in the form of the not-for-profit ICGA Foundation.

The USA**-**National Cancer Institute’s (US-NCI) TCGA has formally agreed to serve as a knowledge partner and will provide technical, operational, troubleshooting, and quality assurance expertise to the ICGA. The Institute of Cancer Research (ICR), UK has formally agreed to help generate and analyze the data and share the data in real-time in a public database.

## The 2^nd^ TCGA 2020 conference: planning the ICGA

The 1^st^ TCGA conference organized in 2019 in India with the theme of “Multi-omics Studies in Cancer: Learnings from The Cancer Genome Atlas (TCGA)”. In addition to discussing the global trends in multi-omics research, the conference proceedings were focused on the need for developing a national mission program for multi-omics profiling of Indian cancer. These discussions laid the foundation of the ICGA project.

With this background, the 2^nd^ TCGA 2020 conference was organized in a virtual and online format to discuss these five main domains of the ICGA project. Highlighting governmental support for the conference and the ICGA, the Hon’ble Dr Harshavardhan (Minister of Science and Technology, Health and Family Welfare and Earth Science, Government of India, opened the conference and Prof Shekhar Mande (Director General CSIR, GoI) presented the conference keynote address. In his keynote address, Dr. Jean Claude Zenklusen (Director, TCGA, US-NCI) shared his experiences of the genesis and implementation of TCGA and highlighted the need for a multi-disciplinary team approach for success of the ICGA.

There was strong multidisciplinary interest in the conference, with over 1500 delegates representing basic science, translational and clinical research, genomics, bioinformatics, biostatistics, and computational science and biology from academia, clinical medicine, industry, and governmental agencies. Delegates attended remotely from 36 countries around the world.

The event (December 3–5, 2020) was held over three days: (1) a one-day hands-on pre-conference workshop, where TCGA experts mentored attendees on various aspects of TCGA data mining; (2) conference presentations discussing global trends in multi-omics cancer research, particularly with respect to the technical aspects of clinical multi-omics studies and applications of precision oncology in the clinic; and (3) a full day of discussion between key ICGA stakeholders on the vision, design, and implementation of the ICGA with regards to common standard operating procedures, operations, logistics, and governance models. The conference sessions were planned around the central theme of building a culture of team science for undertaking mega-cancer research projects in India and neighboring countries.

The 2^nd^ TCGA conference laid the foundation for the ICGA, and there was a wide consensus that the ICGA consortium will unite diverse expertise in cancer research in India and beyond. Given the high prevalence of breast cancer and unanswered questions around clinicopathological features of the disease in Indian women, the ICGA will begin its efforts with the large-scale multi-omics profiling of breast cancer patients in India. With this focus, the ICGA will develop and validate all protocols and ethical guidelines for subsequent sharing with the entire consortium to ensure uniform data generation and quality. It is anticipated that the economy of scale of the meta-analysis of all cancer data generated in India will help deliver cost-effective cancer research and accelerate bench-to-bedside translation to significantly positively impact the overall cancer burden in the country.

In an encouraging development, and motivated by the ICGA project, the Government of Bangladesh has also announced the creation of a Bangladesh Cancer Genome Project (BCGP) modeled on the TCGA and ICGA programs. This partnership will see two parallel large-scale cancer genomic projects span the Indian sub-continent to cover large populations in South Asia.

The 2^nd^ TCGA 2020 Conference brought together the global cancer research community through a common goal to accelerate multi-disciplinary, collaborative, and nationwide initiatives in South Asia, which promises to transform clinical cancer research across the region. The 3^rd^ TCGA Conference in 2021 will report progress on the ICGA and BGCP projects. Overall, these efforts are anticipated to deliberate on the emerging trends in precision oncology in the developing world.

## Funding declaration

The conference proceedings were partially funded by Bajaj Auto Ltd and DBT-Wellcome Trust India Alliance.

## Conflicts of interest statement

The authors have no conflicts of interest.
